# Novel Transfer Learning Approach for Medical Imaging with Limited Labeled Data

**DOI:** 10.3390/cancers13071590

**Published:** 2021-03-30

**Authors:** Laith Alzubaidi, Muthana Al-Amidie, Ahmed Al-Asadi, Amjad J. Humaidi, Omran Al-Shamma, Mohammed A. Fadhel, Jinglan Zhang, J. Santamaría, Ye Duan

**Affiliations:** 1 School of Computer Science, Queensland University of Technology, Brisbane, QLD 4000, Australia; jinglan.zhang@qut.edu.au; 2 AlNidhal Campus, University of Information Technology & Communications, Baghdad 10001, Iraq; o.al_shamma@uoitc.edu.iq; 3 Faculty of Electrical Engineering & Computer Science, University of Missouri, Columbia, MO 65211, USA; mkidn6@mail.missouri.edu (M.A.-A.); aaabc2@mail.missouri.edu (A.A.-A.); duanye@missouri.edu (Y.D.); 4 Control and Systems Engineering Department, University of Technology, Baghdad 10001, Iraq; Amjad.J.Humaidi@uotechnology.edu.iq; 5 College of Computer Science and Information Technology, University of Sumer, Thi Qar 64005, Iraq; Mohammed.a.fadhel@uoitc.edu.iq; 6 Department of Computer Science, University of Jaén, 23071 Jaén, Spain; jslopez@ujaen.es

**Keywords:** deep learning, transfer learning, medical image analysis, convolution neural network (CNN), machine learning

## Abstract

Deep learning requires a large amount of data to perform well. However, the field of medical image analysis suffers from a lack of sufficient data for training deep learning models. Moreover, medical images require manual labeling, usually provided by human annotators coming from various backgrounds. More importantly, the annotation process is time-consuming, expensive, and prone to errors. Transfer learning was introduced to reduce the need for the annotation process by transferring the deep learning models with knowledge from a previous task and then by fine-tuning them on a relatively small dataset of the current task. Most of the methods of medical image classification employ transfer learning from pretrained models, e.g., ImageNet, which has been proven to be ineffective. This is due to the mismatch in learned features between the natural image, e.g., ImageNet, and medical images. Additionally, it results in the utilization of deeply elaborated models. In this paper, we propose a novel transfer learning approach to overcome the previous drawbacks by means of training the deep learning model on large unlabeled medical image datasets and by next transferring the knowledge to train the deep learning model on the small amount of labeled medical images. Additionally, we propose a new deep convolutional neural network (DCNN) model that combines recent advancements in the field. We conducted several experiments on two challenging medical imaging scenarios dealing with skin and breast cancer classification tasks. According to the reported results, it has been empirically proven that the proposed approach can significantly improve the performance of both classification scenarios. In terms of skin cancer, the proposed model achieved an F1-score value of 89.09% when trained from scratch and 98.53% with the proposed approach. Secondly, it achieved an accuracy value of 85.29% and 97.51%, respectively, when trained from scratch and using the proposed approach in the case of the breast cancer scenario. Finally, we concluded that our method can possibly be applied to many medical imaging problems in which a substantial amount of unlabeled image data is available and the labeled image data is limited. Moreover, it can be utilized to improve the performance of medical imaging tasks in the same domain. To do so, we used the pretrained skin cancer model to train on feet skin to classify them into two classes—either normal or abnormal (diabetic foot ulcer (DFU)). It achieved an F1-score value of 86.0% when trained from scratch, 96.25% using transfer learning, and 99.25% using double-transfer learning.

## 1. Introduction

The deep learning (DL) computing paradigm has been deemed the gold standard in the medical image analysis field. It has been exhibiting excellent performance in several medical imaging areas, such as pathology [1], dermatology [2], radiology [3,4], and ophthalmology [5,6], which are the most competitive fields requiring human specialists. The recent approaches within DL being adapted to the direction of clinical alteration commonly depend on a large volume of highly reliable annotated images. Low-resource settings generate different issues, such as gathering highly reliable data, which turn out to be the bottleneck for advancing deep learning applications.

Learning from limited labeled images is a primary concern in the field of medical image analysis using DL since the image annotation process is cost-effective and time-consuming. On the other hand, DL requires a large number of medical images to perform well. Then, transfer learning (TL) has been proposed in this paper to overcome this challenging issue.

Transfer learning (TL) represents the key to success for a variety of effective DL models. Initially, these models are pretrained using a source dataset. They are then fine-tuned using the target task. It has been revealed to be an efficient method when there is a lack of target data. This is commonly found in medical imaging due to the difficulty of collecting medical image datasets. The ILSVRC-2012 competition of ImageNet [7] is the most well-known pretraining dataset and has been extensively utilized to improve the performance of image processing tasks such as segmentation, detection, and classification [8,9,10].

**Figure 1:**
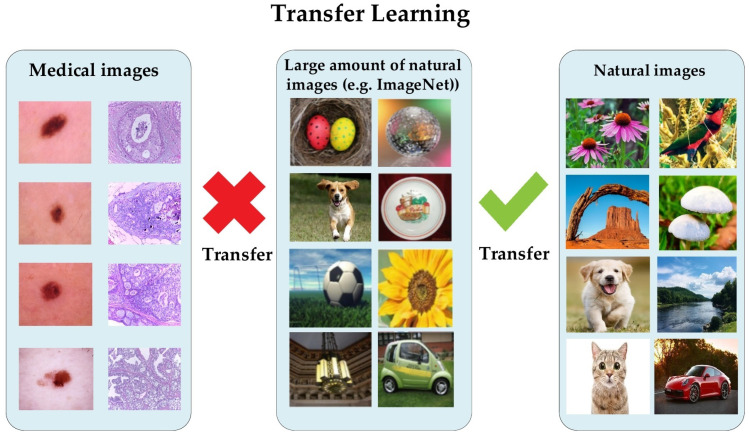
The difference in transfer learning (TL) between natural and medical images.

Conversely, it has been proven that a lightweight model trained from scratch on medical images performed nearly the same as a pretrained model on the ImageNet dataset [11]. The natural images of ImageNet are different from medical images in several aspects, regarding shapes, colors, resolution, and dimensionality (see Figure 1). Recently, Alzubaidi et al. analyzed the benefits of using pretrained models and proved that they are limited in terms of performance improvement dealing with medical images. The authors empirically showed that in-domain TL can improve the performance against the pretrained ImageNet models [12,13,14]. Moreover, it is unnecessary to utilize deeply elaborated models in order to achieve successful results tackling binary classification tasks.

In recent years, there has been significant growth in the amount of unlabeled medical image data facing several tasks. To take advantage of this, we propose transferring the learning knowledge from a large amount of unlabeled medical image data to the small amount of labeled image data of the target dataset. The proposed approach offers several benefits; (i) shortening the annotation process, (ii) benefitting from the availability of large unlabeled medical imaging datasets, (iii) reducing the effort and cost, (iv) guaranteeing that the deep learning model learns the relevant features, and (v) the ability to learn effectively with a small amount of labeled medical images. To prove the effectiveness of the proposed approach, we adopted in this paper two challenging medical imaging scenarios dealing with the skin and breast cancer.

One of the deadliest and fastest-spreading cancers in the world is skin cancer. Seventy-five percent of skin cancer patients die every year [15,16,17,18], while in the USA, there is a risk of skin cancer in every five people who mostly have pale skin and live in an extremely sunny area [15]. In 2017, more than 87,000 emerging cases of melanoma are expected to be diagnosed in the USA [18]. In Australia, 1520 people died from melanoma and 642 from non-melanoma in 2015. Early and accurate diagnosis of skin cancer can save a lot of people’s lives through early treatment [15,17,19,20].

On the other hand, breast cancer is not less dangerous than skin cancer. It is the leading cause of death for women around the world [21,22]. In 2018, the World Health Organization stated that, in its estimation, invasive breast cancer caused about 627,000 women to die. This number represents about 15% of all women cancer-related deaths. Globally, the rates of breast cancer are still progressively growing in most countries according to 2020 statistics [23].

Briefly, the proposed approach is suitable for any medical imaging task that has plenty of unlabeled images with limited labeled images. Furthermore, it can also help to enhance the performance of tasks in the same domain, e.g., the pretrained model on histopathology images of the breast can be used for any task that uses the same image format, such as colon cancer and bone cancer. Another example of its application is that in which the pretrained model on skin cancer images can be used to improve the performance of any task related to skin diseases. To prove this, we fine-tuned the pretrained skin cancer model to be trained on feet skin images to classify them into two classes: either normal or abnormal (diabetic foot ulcer (DFU)). For medical imaging classification tasks with limited unlabeled and labeled images, we also present the concept of double-transfer learning on the diabetic foot ulcer (DFU) classification task. It is a technique that uses the pretrain model of the skin cancer task and then trains it on a small number of unlabeled feet images to improve the learned features. After that, the model is fine-tuned to train on the small labeled dataset. This approach helps in the tasks that have a small number of unlabeled and labeled images.

Our work reveals interesting contributions, as follows:

1. We demonstrate that the proposed approach of transfer learning from large unlabeled images can lead to excellent performance in medical imaging tasks.
2. We introduce a hybrid DCNN model that integrates parallel convolutional layers and residual connections along with global average pooling.
3. We train the proposed model with more than 200,000 unlabeled images of skin cancer and then fine-tune the model for a small dataset of labeled skin cancer to classify them into two classes, namely benign and malignant. We also train the proposed model with more than 200,000 unlabeled hematoxylin–eosin-stained breast biopsy images. We then fine-tune the model for a small dataset of labeled hematoxylin–eosin-stained breast biopsy images to classify them into four classes: invasive carcinoma, in situ carcinoma, benign tumor, and normal tissue.
4. We apply several data augmentation techniques to overcome the issue of unbalanced data.
5. We combine all contributions to improve the performance of two challenging tasks: skin and breast cancer classification tasks. In the skin cancer classification, the proposed model achieved an F1-score value of 89.09% when trained from scratch and 98.53% with the proposed approach on SIIM-ISIC Melanoma Classification 2020. The proposed model achieved an F1-score value of 85.29% when trained from scratch and 97.51% with the proposed approach ICIAR-2018 dataset for the breast cancer classification task. The results proved that the first and second contributions are effective in the medical imaging tasks.
6. We utilize the pretrained skin cancer model to improve the performance DFU classification task by fine-tuning it to train on feet skin images to classify them into two classes, either normal or abnormal. It attained an F1-score value of 86.0% when trained from scratch and 96.25% with transfer learning.
7. We introduce another type of transfer learning besides the proposed approach, which is double-transfer learning. We achieved an F1-score value of 99.25% with the DFU classification task using this technique.
8. We test our model trained with the double-transfer learning technique on unseen DFU test set images. Our model achieved an accuracy of 97.7%, which proves that our model is robust against overfitting.

The rest of the paper is organized as follows: Section 2 describes the literature review. Section 3 explains the materials and methods. Section 4 reports the results. Lastly, Section 5 concludes the paper.

## 2. Literature Review

This section is divided into two sections. The first one describes TL within medical imaging and introduces the drawbacks of previous TL methods in the state-of-the-art. The second section presents the developments of CNN models and the problems that we dealt with when designing our proposed model.

### 2.1. Transfer Learning for Medical Imaging

Transfer learning is a commonly utilized technique when developing medical imaging models due to a lack of training data. One of the first ideas to use transfer learning was to adopt pretrained models of the ImageNet dataset instead of training from scratch [9,10,24]. Nevertheless, there is a significant difference between the characteristics of medical images and the natural image of datasets, such as ImageNet [7] (see Figure 1). Medical images of a specific field frequently have standardized views, such as the features of relevant tasks tending to have limited texture variants or small patches rather than high-level semantic features. A high-resolution is commonly significant, and images are often grayscale, for example, X-ray images. Raghu et al. [11] performed empirical experiments using two large medical datasets—retinal fundus [6] and a chest X-ray (CheXpert [25])—to improve understanding of the ImageNet tradeoffs, including TL. These two experiments demonstrated that the domain variance between medical and natural images restricts TL. More specifically, the performance was not considerably enhanced when starting training with the pretrained weights of ImageNet around a variety of architectures. Moreover, by utilizing randomly initialized smaller architectures, carefully crafted, the achieved performance was similar to that of the larger pretrained models of ImageNet. These findings agreed with the experiments’ performance, as conducted by Neyshabur et al. [26], where they proposed that the important contributions to performance advances using TL are due to feature reuse and the learning of low-level image statistics.

Recent works proposed another highly effective technique, known as in-domain pretraining, alongside the previously discussed transfer learning [13,27,28]. For instance, Heker and Greenspan [28] introduced the utilization of trained weights using an in-domain dataset for liver-segmentation purposes rather than the utilization of initialized weights from a model trained using ImageNet. They stated that pretraining using a separate dataset of liver imaging gives an enhanced performance better than a pretrained ImageNet. On the same line, Alzubaidi et al. [14] trained their model on histopathology images of colon cancer. Then, they fine-tuned the model for the histopathology images of breast cancer. They found that this technique improved the performance more than natural images. Although this type of transfer learning showed good results, it still needs to be trained with labeled data to obtain a pretrained model. It is well-known that labeling medical data is time-consuming and very costly. Therefore, It is necessary to tackle these issues with a new approach, which we propose in this paper. Our approach is based on using large unlabeled image data of a specific task then fine-tunes the model for a small labeled dataset for the same task.

### 2.2. Convolutional Neural Networks

Deep learning has become an incredibly popular machine learning algorithm in recent years due to the vast growth and evolution of the big data field [29,30]. It is still in continuous inspirational development regarding the novel performance of several machine learning tasks [31,32,33,34], and it has simplified the improvement of many learning fields [35,36]. Deep learning defines an end-to-end machine learning method. It has the ability to extract features automatically. Both steps of feature exaction and classification can be accomplished in one shot with deep learning. However, in traditional machine learning, there is a series of steps to achieve the classification starting by preprocessing, feature extraction, feature selection, and then classification. Deep learning can extract complex features that help to distinguish between classes. Moreover, it is able to handle a large amount of data, unlike traditional machine learning. One of the key successes of deep learning is convolutional neural networks (CNNs). A CNN has the ability to effectively recognize higher-level objects and to learn the hierarchical levels of representations from a low-level input vector. In addition, it has the ability to gradually extract higher image representations after every single layer, and lastly, it identifies the image [37,38].

CNN is the best choice for image and video classification due to its hierarchical architecture and its success in several complex medical image applications [39,40,41]. It is also employed in different areas, such as drug discovery, natural language processing (NLP), etc. A CNN is composed of a sequence of layers starting with a series of convolutional and subsampling layers. The fully connected layer and Softmax function layer comes at the end. A sequence of multi-layer convolution gradually performs additional refined-feature extraction in each layer starting from the beginning (input) layer to the ending (output) layer. Following feature extraction using the convolutional layers, the classification task is performed using the fully connected layer. The input of the CNN is an image of 2-D n × n pixels. Every single layer is composed of collections of 2-D neurons, known as kernels or filters. Regarding neural networks, the CNN neurons differ from others in terms of their connection. The connection of neurons between two adjacent layers does not follow the all-to-all method. Alternatively, they are connected to the spatially fixed-size mapped and partly overlapped neurons in the feature map of the previous layer. This area in the input is called the local receptive field. When the number of connections is reduced, this leads to a reduction in the overfitting chance and the training time. Note that all the neurons in a kernel are connected to a similar number of neurons in the preceding input layer. In addition, they are restricted to obtain a similar series of biases and weights. These features decrease the memory requirements and accelerate the learning of the network. Therefore, every single neuron of a specified kernel searches the same form in other input-image parts. The pooling layers decrease the network size. Furthermore, these layers efficiently decrease the network sensitivity to image distortions, scales, and shifts along with shared weights and local receptive fields (inside the same filter) [38]. Pooling is frequently achieved using local averaging filters or a max/mean operation. The CNN ending layers perform the classifications since the neurons within these layers are fully connected. The implementation of the deep CNN can be achieved with multi-series of weight-share convolutional layers and pooling layers. High-quality representations are the result of deep CNN, at the same time, preserving locality, lessened parameters, and invariance fewer variants in the input image [42]. In recent years, a huge development of complex CNN models has been shown [38,43,44], which started in 2012 with the AlexNet model. In 2014, the Visual Geometry Group proposed the VGG-16 network, which is still applied frequently. VGG-19, which has nineteen layers of learnable parameters, is the newer version of VGG-16. Both versions have considerably similar representation power. GoogLeNet, which is also known as the Inception network, is another well-liked network. It derives its highest power from using a unique type of layer, known as the inception block/layer. The authors of this network updated it four times. Each time, the newer version has slightly enhanced representation power (under a specific viewpoint). Another well-known network that has the ability of DL with more than 100 layers in its models is called ResNet. Residual learning is the concept that ResNet is based upon. Due to its ability to build very deep networks, ResNet comes highly recommended by the pattern recognition community. A more compact network called DenseNet is another network similar to ResNet that utilizes residual learning insight to obtain similar representation power. These models were designed to classify 1000 classes on natural images of the ImageNet dataset. It is unnecessary to employ these very deep models for two or three class classification tasks. Moreover, these models have fixed input sizes. It is difficult to handle large-sized images. To overcome these issues, we designed our proposed model, which considers the advantages of state-of-the-art architectures. We added several improvements to tackle several issues. These improvements, including parallel convolutional layers and short and long connections for better feature extraction, avoid adding a pooling layer after each convolutional layer, which could lead to losing significant features in the learning stage. Instead, we employed global average pooling at the end, which helps to prevent overfitting. Batch normalization layer and ReLU were added after each convolutional layer to speed up the training process and to prevent the gradient-vanishing problem.

## 3. Materials and Methods

This section consists of six parts: the proposed approach, the datasets, the data augmentation techniques, the proposed model, the training scenario, and double-transfer learning.

### 3.1. The Proposed Approach

**Figure 2:**
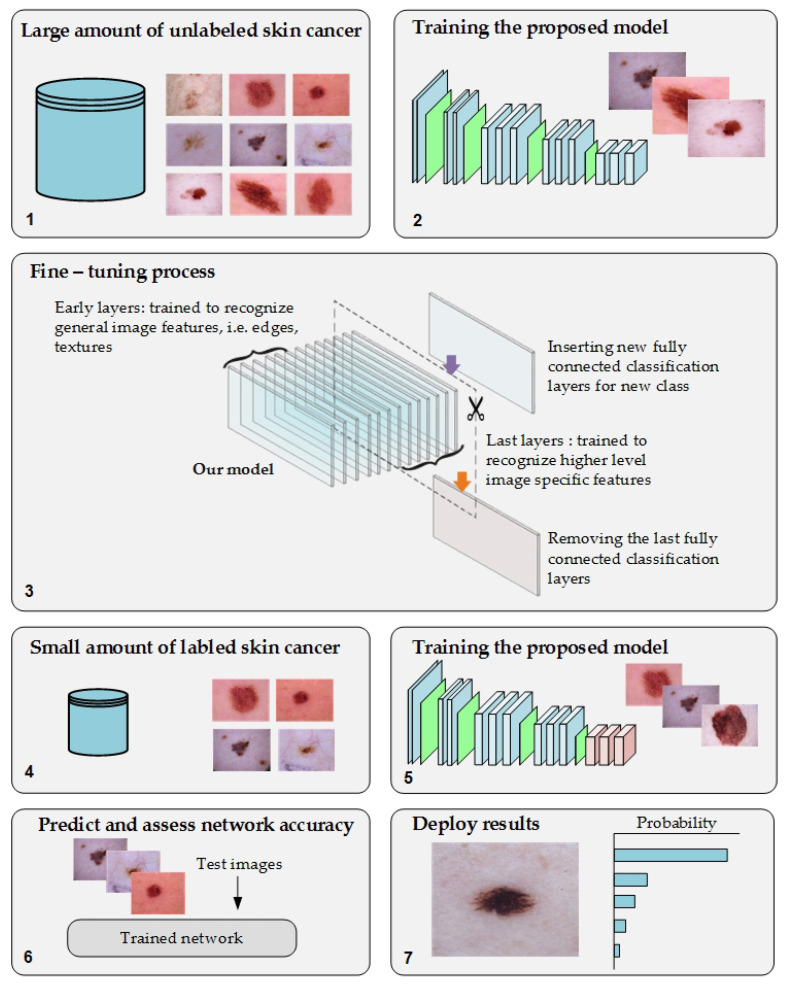
The workflow of the proposed approach.

We propose a novel approach of TL to overcome the issues of transfer learning from pretrained models of the ImageNet dataset to medical imaging tasks and the annotation process of medical images. Moreover, it will help to address the issue of the lack of training in medical imaging tasks. The proposed approach was based on training the DL model on a large number of unlabeled images of specific tasks since there is significant growth in the unlabeled medical images. After the fine-tuning process, the model was trained on a small, labeled dataset for that same task. Figure 2 depicts the workflow of the proposed approach.

This approach guarantees that the model will learn the relevant features and minimize the effort of the labeling process. To test the proposed approach, we employed two challenging medical imaging scenarios dealing with skin and breast cancer classification tasks. Both tasks have a large archive of images. To benefit from that, we used an archive of these tasks to improve the performance of recent datasets of the same tasks. In this paper, we used more than 200,000 unlabeled images of skin cancer to train the proposed model. The model was fine-tuned by considering a small dataset of labeled skin cancers to classify them into two classes, namely benign and malignant. Additionally, the proposed model was trained using more than 200,000 unlabeled hematoxylin–eosin-stained breast biopsy images and, next, fine-tuning the model for a small dataset of labeled hematoxylin–eosin-stained breast biopsy images to classify them in four classes: invasive carcinoma, in situ carcinoma, benign tumor, and normal tissue.

The main purpose of training the model with unlabeled images is to improve the learning stage of the model. Thus, the convergence of weights will be attainable. Since the purpose is for learning and not for the classification stage, the labels do not necessarily need to be accurate. Therefore, we assigned random labels such as giving each dataset in the source dataset the name of the dataset as a label.

The proposed approach is not limited to skin and breast cancer classification tasks. It can be utilized for any medical imaging task that has a large number of unlabeled images with a small number of labeled images. It can also improve the performance of medical imaging tasks in domain, as explained in the double-transfer learning section.

### 3.2. Dataset

This part consists of two main subparts. The first subpart describes the source datasets of both the skin and breast cancer tasks. The purpose of these datasets is to generate a pretrain model for the target dataset. The second subpart describes the target dataset of both the skin and breast cancer tasks.

#### 3.2.1. Source Dataset

**Figure 3:**
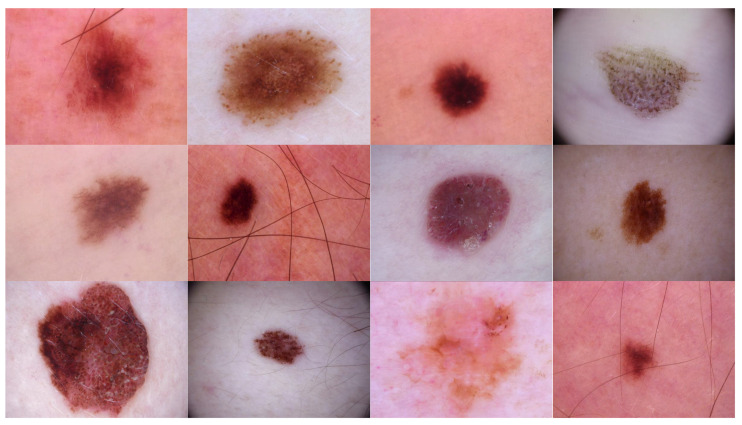
Samples of source domain dataset of skin cancer.

**Figure 4:**
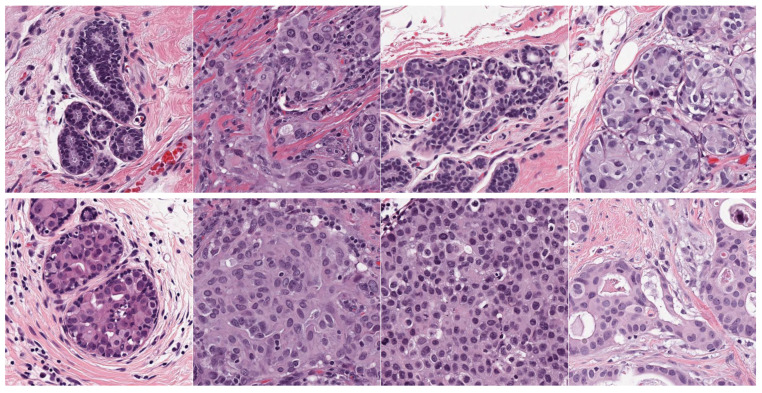
Samples of the source domain dataset of breast cancer.

1. Source domain dataset of skin cancer: The main source of this dataset is the ISIC Challenges datasets (2016, 2017, 2018, 2019, and 2020) of skin lesion classification tasks [20,45,46,47,48,49]. The total number of images is 81,475. We added 100 melanoma and 70 naevus images of the MED-NODE dataset [50]. The last source is the Dermofit dataset, which consists of 1300 skin lesion images of malignant and benign [51]. All collected images were duplicated to more than 200,000 images by using data augmentation techniques. Figure 3 shows some samples of the dataset.
2. Source domain dataset of breast cancer: we collected the histopathology images of breasts from various sources. The first source is the BreakHis dataset [52]. This dataset is composed of 9109 microscopic images of breast tumor tissue with a size of 700 × 460 pixels. Each image was divided into two images of 350 × 230 and then resized to 512 × 512. The second source is the histopathological microscopy image dataset of IDC [53]. It consists of 922 images with sizes of 2100 × 1574 and 1276 × 956 pixels. The third source is the breast cancer dataset, which is composed of 537 H&E-stained histopathological images with a size of 2200 × 2200 pixels [54]. The fourth source is the BreCaHAD dataset [55]. This dataset consists of 162 breast cancer histopathology images with a size of 1360 × 1024 pixels. The fifth source is the SPIE-AAPM-NCI BreastPathQ dataset [56], which is composed of 2579 patches of histopathology images of the breast. These patches were extracted from 96 images with a size of 512 × 512. The sixth source is the image dataset from the bioimaging 2015 breast histology classification challenge [57]. There are 249 images with a size of 2040 × 1536 pixels. All the images of sources two to six were divided into 12 nonoverlapping patches of 512 × 512 sizes. The total was 50,314 breast histology patches. All collected patches were duplicated to more than 200,000 images by using data augmentation techniques. It is worth mentioning that we cropped the images to 512 × 512 to fit the input size of the model. This guarantees that the proposed model detects the features that define the nucleus-localized organization and the overall tissue architecture required to distinguish between the classes. Conversely, taking a small size could result in a loss of information associated with the same assigned class of the entire image. Figure 4 shows some samples of the dataset.

#### 3.2.2. Target Dataset

**Figure 5:**
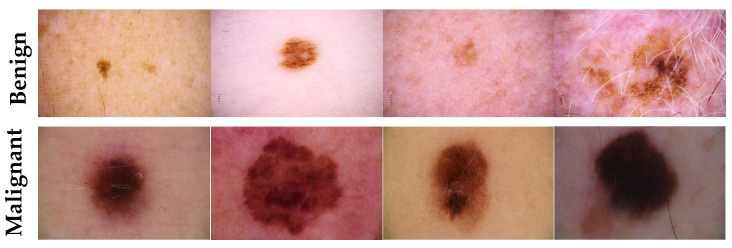
Samples of the target domain dataset of skin cancer.

**Figure 6:**
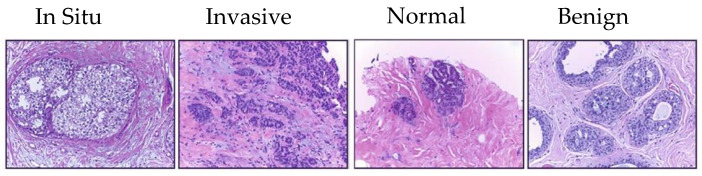
Samples of target domain dataset of breast cancer.

1. Target dataset of skin cancer: The proposed model has been trained and tested after TL from the source skin cancer dataset on the SIIM-ISIC 2020 dataset [49]. The latter consists of 33,000 skin lesion samples classified into two categories: benign and malignant. We took part of the dataset, which was 9000 images, for the benign class, and the rest was added to the source dataset, with only 584 samples of the malignant class. To tackle the issue of imbalanced classes, we performed several data augmentation techniques on malignant class samples. The reason behind taking part of the dataset is to check how the proposed model with the proposed approach can perform when it trains on a small dataset. The part taken from the dataset was divided into 80% for training and 20% for testing. We resized all images to 500 × 375 to minimize the computational cost and to speed up the training process. Figure 5 shows some samples of the dataset.
2. Target dataset of breast cancer: the ICIAR-2018 (BACH 2018) Grand Challenge provided this dataset [58]. The images were uncompressed and in high-resolution mode (2040 × 1536 pixels). They consisted of H&E-stained breast histology microscopy images and were labeled as normal tissue, benign lesion, in situ carcinoma, or invasive carcinoma (see Figure 6). The labeling stage was achieved by two medical steps, utilizing identical acquisition cases, with an enlargement of 200. A total of 400 images were used (100 samples in each class). These images were chosen so that the pathology recognition could be independently distinguished from the visible organization and the tissue structure. The dataset was divided into 300 images for the training set and 100 for the testing set. The original image was divided into 12 nonoverlapping patches of 512 × 512 pixels in size.

### 3.3. Augmentation Techniques

In this paper, we applied several data augmentation techniques to overcome the issue of unbalanced data and to increase the training set. These techniques are data-space solutions for any limited-data problem. Data augmentation incorporates a collection of methods that improve the attributes and size of training datasets. Thus, DL networks can perform better when these techniques are employed. Furthermore, these techniques help prevent the issue of overfitting. Next, we list some data augmentation techniques that we employed in this paper.

1. Random rotation between 45 and 315 degrees
2. Crop the region of interest (in the task of the skin and DFU)
3. Random brightness, random contrast
4. Zoom
5. Perform an erosion effect. The erosion is a morphological effect applied on the image that shrinks the object in the image and can be mathematically defined as in Equation (1):
  (1) $$A\Theta B=\left\{\left.z\in E|B_{z}\subseteq A\right\}\right.$$
  where *A* is the image to be eroded and *B* is the structuring element.
6. A dilation is an inverse of erosion that is performed on an image and can increase the area of the object. Here, we perform a double dilation effect. The dilation process can be described mathematically as in Equation (2):
  (2) $$A⊕B=\left\{\left.z|\left(\hat{B}\right)z\cap A\right\}\right.$$
7. Add Gaussian Noise.

### 3.4. The Proposed Model

Our proposal is based on an effective DCNN model that combines several creative components to solve many issues, including better feature extraction, gradient-vanishing problem, and overfitting. These components can be summarized as follows:

- Traditional convolutional layers at the beginning of the model to reduce the size of input images
- Parallel convolutional layers with different filter sizes to extract different levels of features to guarantee that the model learns the small and large features
- Residual connections and deep connections for better feature representation. These connections also handle the issue of gradient vanishing.
- Batch normalization to expedite the training process
- A rectified linear unit (ReLU) does not squeeze the input value, which helps minimize the effect of the vanishing gradient problem.
- Dropout to avoid the issue of overfitting
- Global average pooling makes an extreme dimensionality reduction by transforming the entire size to one dimension. This layer helps to reduce the effect of overfitting.

**Figure 7:**
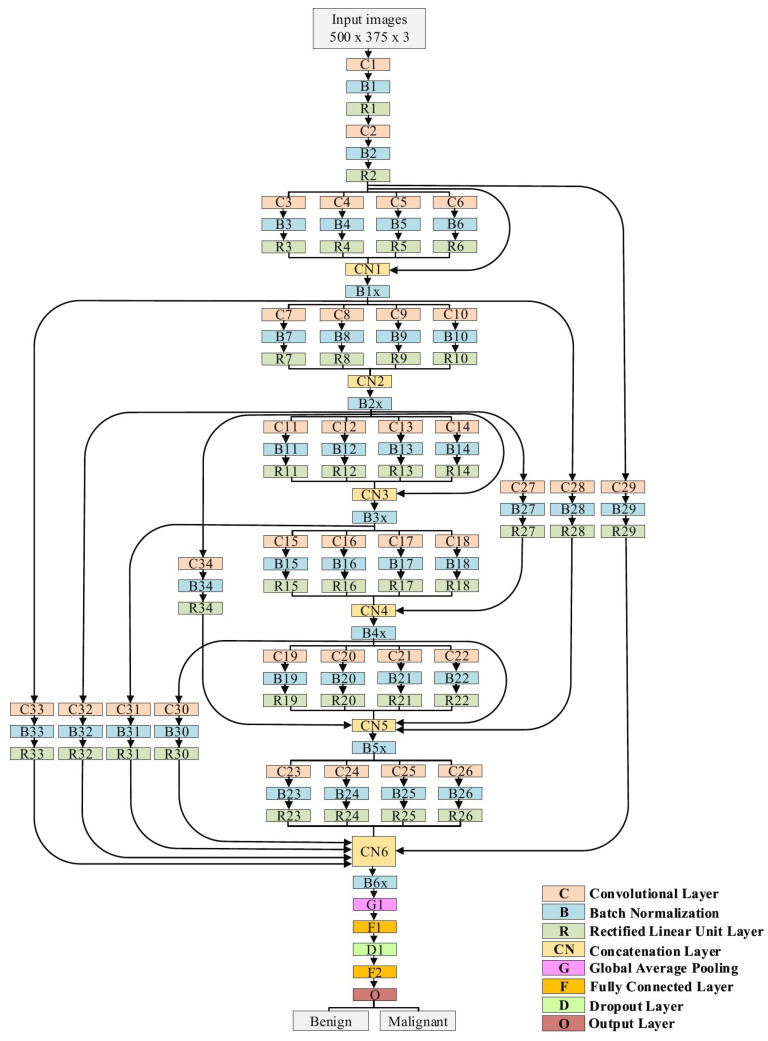
The architecture of the proposed model.

The proposed model is explained in detail in Table 1 and Figure 7. In the case of the skin cancer classification scenario, the input size of the proposed model is 500 × 375. Regarding the breast cancer scenario, the input size is modified to 512 × 512. The model starts with two traditional convolutional layers working in sequence. The first one has a filter size of 3 × 3, while the second convolution has a filter size of 5 × 5. Both convolutional layers are followed by BN and ReLU layers. We avoided utilizing small filters, such as 1 × 1, at the beginning of the model to prevent losing small features, which in the results, will perform as a bottleneck. Six blocks of parallel convolutional layers come after the traditional convolutional layers. Each block comprises four parallel convolutional layers with four distinct filter sizes (1 × 1, 3 × 3, 5 × 5, and 7 × 7). The output of these four layers is integrated into the concatenation layer to move to the following block. All convolutional layers in all six blocks are followed by BN and ReLU layers. There are ten connections between the blocks. Some of them are short and others are long, with a single convolutional layer. These connections maintain the model’s ability to have different levels of features for the purpose of better feature representation. Both parallel convolutions and the connections are extremely important for gradient propagation as the error can backpropagate from multiple paths. Finally, two fully connected layers are adopted with one dropout layer between them. Softmax is employed to finalize the output. In total, our proposed model consists of 34 convolutional layers.

### 3.5. Training Scenario

**Figure 8:**
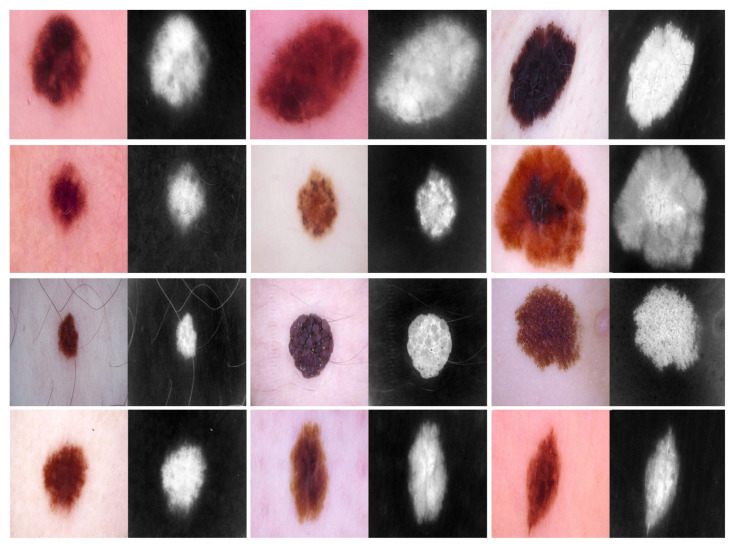
Learned filters from the first convolution layer of the model trained on the skin cancer datasets, single filter of single image. The color image is the original, and the gray-scale image is the filter.

The training procedure of the proposed model is achieved in the following two phases:

- Phase #1: Training the proposed model from scratch using the target dataset of the skin cancer classification task.
- Phase #2: Training our model on source domain dataset of skin cancer. We then fine-tune the model for the target dataset of the skin cancer classification task (see Figure 2). Figure 8 shows the learned filters from the first convolutional layer. The learned filters from the first convolutional layer indicate that our model learned excellent features from the beginning.

**Figure 9:**
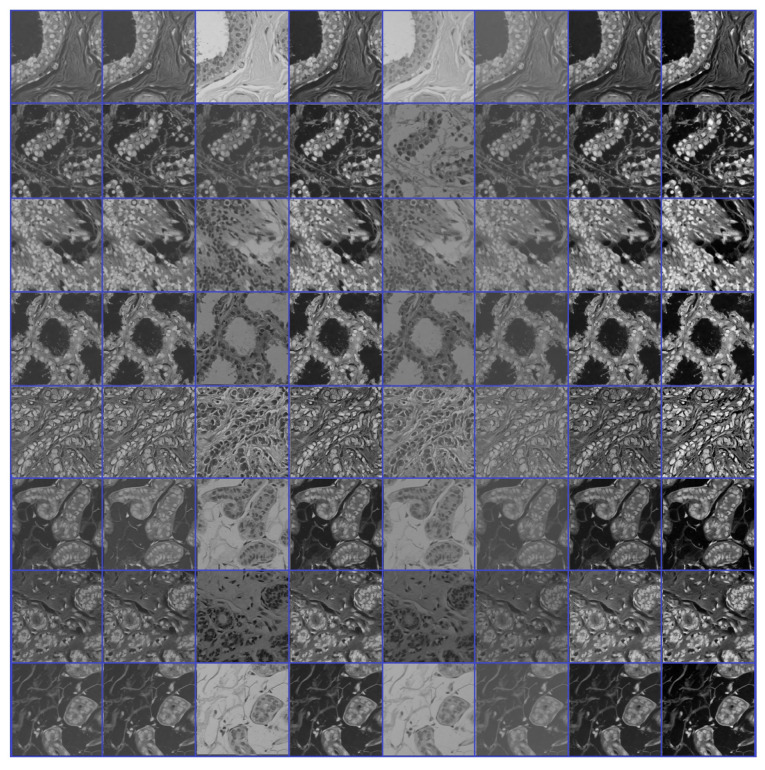
Learned filters from first convolution layer of the model trained on the ICIAR-2018 dataset [58], multiple filters of multiple images.

**Figure 10:**
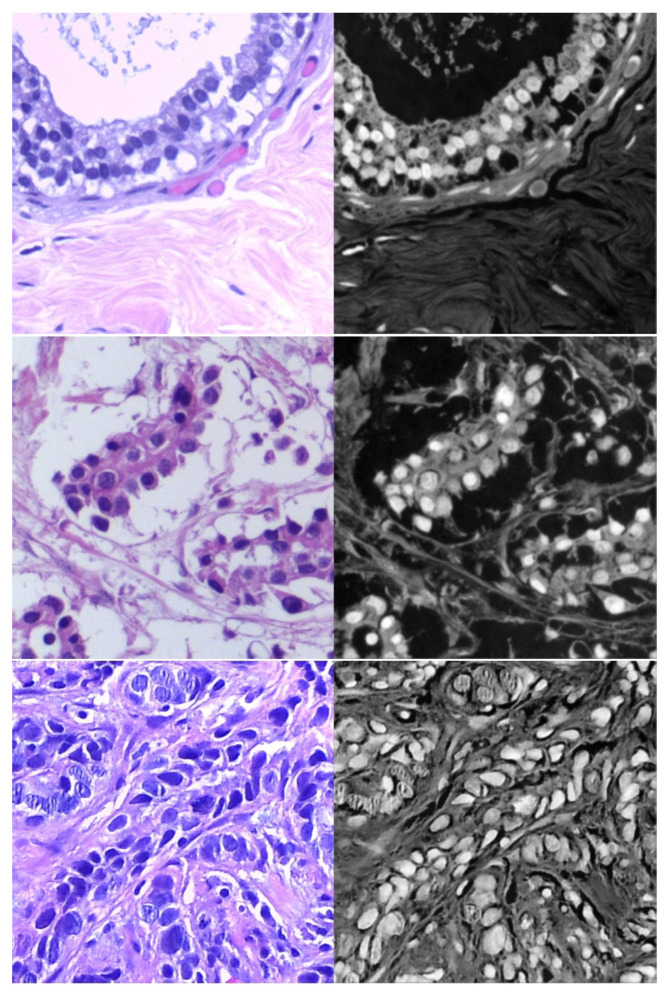
Learned filters from first convolution layer of the model, single filter of single image. The color image is the original, and the gray-scale image is the filter.

We repeated phases #1 and #2 for the breast cancer classification task with respect to the breast cancer datasets (source + target). Figure 9 and Figure 10 show the learned filters from the first convolutional layer. The training options are listed as follows:

1. Stochastic gradient descent with a momentum set to 0.9
2. The mini-batch size was 64 and MaxEpochs was 100.
3. The learning rate was initially set to 0.001.

We ran our experiments on MATLAB 2020 software and a processor Intel (R) Core TM i7-5829K CPU at 3.30 GHz, 32 GB RAM, and 16 GB GPU.

### 3.6. Double-Transfer Learning Technique

**Figure 11:**
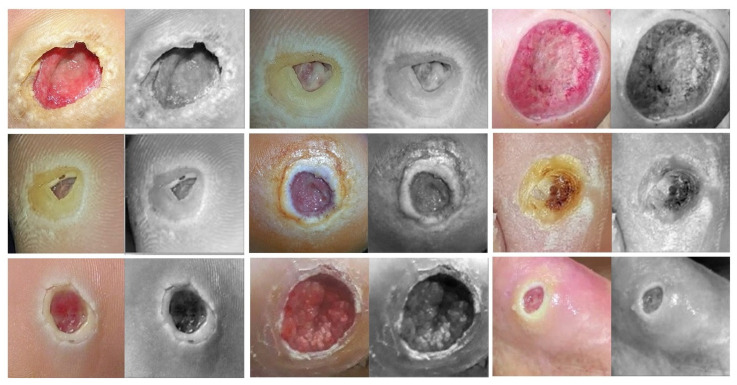
Learned filters from first convolution layer of the model trained on the DFU dataset [59], Single filter of single image. The color image is the original, and the gray-scale image is the filter.

**Figure 12:**
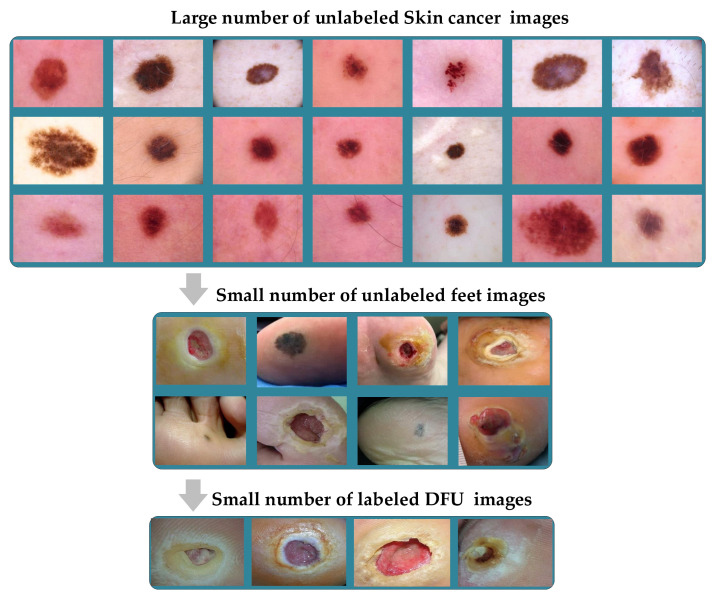
Double-Transfer learning technique with the diabetic foot ulcer (DFU) task.

The pretrained models on skin cancer and breast cancer are not limited to these tasks. They can also aid in further enhancing the performance of medical imaging tasks in the same domain. For example, the pretrained model on skin cancer images can be used to improve the performance of any task related to skin diseases. To test this, we worked on a DFU classification task. The aim of this task is to classify feet skin into two classes, namely normal (healthy skin) and abnormal (DFU). This experiment is significant for the task of DFU since this task suffers from a lack of images. We accomplished three training phases on the DFU classification task as follows:

1. Phase #1: Training our model from scratch using the DFU dataset [59] that contained two classes, normal and abnormal
2. Phase #2: Fine-tuning the pretrained model of skin cancer task for the DFU classification task and then training it on the DFU dataset [59]. Figure 11 shows the learned filters from the first convolutional layer.
3. Phase #3: First, fine-tuning the pretrained model of skin cancer task to train it on a small number of unlabeled feet skin images. We collected 2000 images of feet skin diseases including DFU from an internet search and part of the DermNet dataset [60]. We increased this number to more than 10,000 using data augmentation techniques. Second, fine-tuning the pretrained model that results from the first step to train it on a small number of labeled DFU images [59]: by doing that, we achieve a double-transfer learning technique. Figure 12 shows the steps of the double-transfer learning technique.

For some of the medical imaging tasks, such as DFU, it is hard to obtain a large number of unlabeled images to train a pretrain model. At the same time, it is significantly harder to obtain labeled DFU images. To the best of our knowledge, there is only one public DFU dataset [61] and a private dataset [59] that we use in this paper.

The pretrained model using either skin cancer task or breast cancer task can behave as a base learned model for other medical imaging tasks in the same domain to obtain excellent learning. Furthermore, with the double-transfer learning technique, it is easy to turn the pretrained model for any medical imaging task in the domain. Both the proposed approach and double-transfer learning can be applied to many medical imaging tasks.

## 4. Results

This section is divided as follows: evaluation metrics, results of the skin cancer classification scenario, results of the DFU classification scenario, and results of the breast cancer classification scenario.

### 4.1. Evaluation Metrics

We evaluated our model based on several evaluation metrics including accuracy, recall, precision, and *F*1-score. These evaluation metrics were calculated based on the calculation of *TN*, *TP*, *FN*, and *FP*. *TN* and *TP* are defined as the number of negative and positive instances, respectively, which are successfully classified. In addition, *FN* and *FP* are defined as the number of misclassified positive and negative instances, respectively. The definition and equation of each evaluation metrics are listed below:

1. Accuracy calculates the ratio of correct predicted classes to the total number of samples evaluated (Equation (3)).
  (3) $$Accuracy=\frac{TP+TN}{TP+TN+FP+FN}$$
2. Sensitivity or recall is utilized to calculate the fraction of positive patterns that are correctly classified (Equation (4)).
  (4) $$Recall=\frac{TP}{TP+FN}$$
3. Precision is utilized to calculate the positive patterns that are correctly predicted by all predicted patterns in a positive class (Equation (5)).
  (5) $$Precision=\frac{TP}{TP+FP}$$
4. *F*1-score calculates the harmonic average between recall and precision rates (Equation (6)).
  (6) $$F1_{score}=2\times \frac{Precision\times Recall}{Precision+Recall}$$

### 4.2. Results of the Skin Cancer Classification Scenario

This experiment was conducted to classify the skin lesions into two categories on the SIIMISIC 2020 dataset, benign and malignant. As reported in Table 2, our model in phase #1 obtained an accuracy of 89.69%, a recall of 85.60%, a precision of 92.86%, and an F-score of 89.09%. Although these results are good, in phase #2, the results are significantly better. Our model in phase #2 obtained 98.57%, 97.90%, 99.18%, and 98.53% for accuracy, recall, precision, and the F-score, respectively. The results in Table 2 indicate that the proposed approach of transfer learning significantly improved the results. To the best of our knowledge, the highest results achieved on the SIIMISIC 2020 dataset are 0.9411 and 0.96317 area under the ROC curve [62,63], respectively.

### 4.3. Results of DFU Classification Scenario

To test the pretrained model of skin cancer on other tasks, we conducted an experiment on DFU classification to distinguish feet skin images as either normal or abnormal, as reported in Table 3. The pretrained model on the skin cancer task improved the result in phase #2 by achieving an accuracy of 96.10%, recall of 97.06%, a precision of 95.45%, and a F-score value of 96.25% compared to phase #1, which achieved 83.18%, 83.03%, 89.21%, and 86.0% for accuracy, recall, precision, and F-score, respectively. On the other hand, the phase #3 results of double-transfer learning outperformed the results of phases #1 and #2. Our model in phase #3 obtained 99.03%, 99.81%, 98.7%, and 99.25% for accuracy, recall, precision, and the F-score, respectively.

Our results in phase #3 of double-transfer learning not only outperformed phase #1 or #2 but also outperformed previous methods that worked on the same dataset, as reported in Table 4. It is worth mentioning that phase #2 also outperformed the previous methods listed in Table 4.

### 4.4. Results of Breast Cancer Classification Scenario

**Figure 13:**
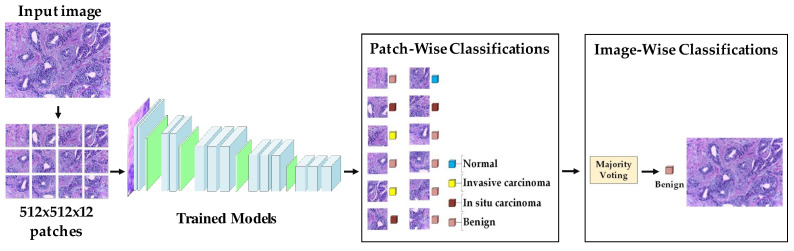
The procedure of evaluation of the breast cancer task.

We performed our experiment on the ICIAR-2018 dataset of hematoxylin–eosin-stained breast biopsy images to classify them into four classes: invasive carcinoma, in situ carcinoma, benign tumor, and normal tissue. There were four steps to obtain the final accuracy of test images. (i) Each image was divided into 12 nonoverlapping patches of 512 × 512; (ii) the probabilities were calculated by the classifier of the trained model; (iii) a majority voting technique was employed, where the patch label, which was the most dominant, was determined to be the image label; and (iv) after accomplishing the results of all the test images, the accuracy was calculated. The procedure of evaluation is shown in Figure 13.

The results in Table 5 exhibit how the proposed approach of transfer learning improved the performance of the breast cancer task by ameliorating the accuracy of 85.29% in phase #1 to 97.51% in phase #2. Additionally, our model in phase #2 surpassed the previous methods, as stated in Table 6.

### 4.5. Performance Evaluation on Unseen DFU Test Set

**Figure 14:**
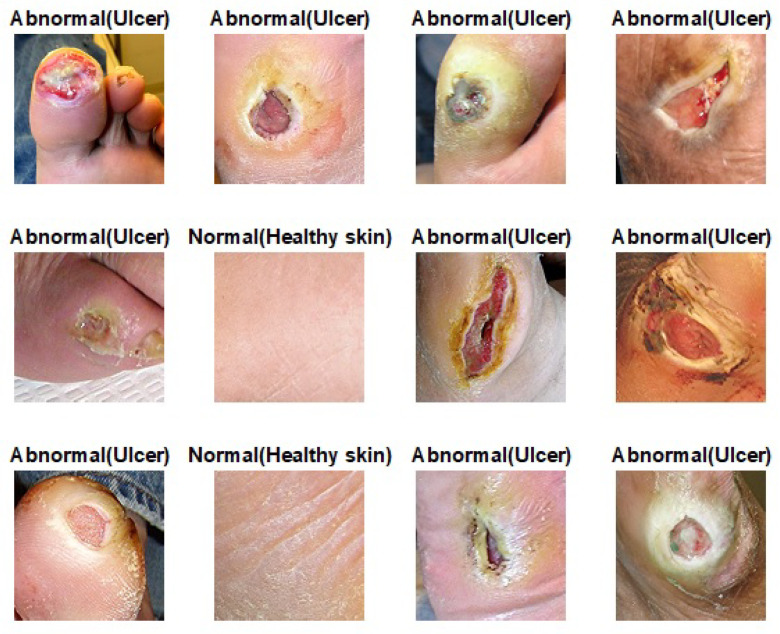
Some samples of prediction of the DFU test set.

To test the robustness of the proposed model against overfitting, we tested it on DFU test set images provided by the Louisiana State University Health Sciences Center, New Orleans, United States [71]. The center provided us with 60 DFU images. We extracted 60 patches of DFU and 60 patches of normal class from the internet search. We tested our model that trained with phase #3 in the section Double-Transfer Learning Technique. Our model achieved an accuracy of 97.7%, a recall of 93.8%, a precision of 100%, and a F-score value of 96.8%. Some of the prediction samples of the DFU test set are shown in Figure 14. The results proved that our model is robust against overfitting, as it tested on unseen images.

## 5. Conclusions

We can conclude by highlighting six major points in this paper. (i) We proposed a novel approach of TL to tackle the issue of the lack of training data in medical imaging tasks. The approach is based on training the DL models on a large number of unlabeled images of a specific task and then fine-tuning the model to train on a small number of labeled images for the same task. (ii) We designed a hybrid DCNN model based on several ideas, including parallel convolutional layers and residual connections along with global average pooling. (iii) We empirically proved the effectiveness of the proposed approach and model by applying them in two challenging tasks, skin and breast cancer. (iv) We utilized more than 200,000 unlabeled images of skin cancer to train the model, and then, we fine-tuned the model for a small dataset of labeled skin cancer to classify them into two classes, namely benign and malignant. We used the same procedure for the breast cancer task to classify histology breast images into four classes: invasive carcinoma, in situ carcinoma, benign tumor, and normal tissue. (v) We achieved excellent results in both tasks. In the skin cancer classification task, the proposed model achieved a F1-score value of 89.09% when trained from scratch and 98.53% with the proposed approach. The proposed model achieved a F1-score value of 85.29% when trained from scratch and 97.51% with the proposed approach for the breast cancer task. (vi) Additionally, we introduced another technique of transfer learning called double-transfer learning. We employed it to improve the performance DFU classification task, and we obtained a F1-score of 99.25%. Lastly, we aimed to use the learned features to improve the performance of other tasks, such as skin cancer segmentation.

## Figures and Tables

**Table 1:** Our model architecture. C = Convolutional layer, B = Batch normalization layer, R = Rectified linear unit layer, CN = Concatenation layer, G = Global average pooling layer, D = Dropout layer, and F = Fully connected layer.

Name of Layer	Filter Size (FS) and Stride (S)	Activations
Input layer	-	500 × 375 × 3
C1, B1, R1	FS = 3 × 3, S = 1	500 × 375 × 32
C2, B2, R2	FS = 5 × 3, S = 2	250 × 188 × 32
C3, B3, R3	FS = 1 × 1, S = 1	250 × 188 × 32
C4, B4, R4	FS = 3 × 3, S = 1	250 × 188 × 32
C5, B5, R5	FS = 5 × 5, S = 1	250 × 188 × 32
C6, B6, R6	FS = 7 × 7, S = 1	250 × 188 × 32
CN1	Five inputs	250 × 188 × 160
B1x	Batch normalization layer	250 × 188 × 160
C7, B7, R7	FS = 1 × 1, S = 2	125 × 94 × 64
C8, B8, R8	FS = 3 × 3, S = 2	125 × 94 × 64
C9, B9, R9	FS = 5 × 5, S = 2	125 × 94 × 64
C10, B10, R10	FS = 7 × 7, S = 2	125 × 94 × 64
CN2	Four inputs	125 × 94 × 256
B2x	Batch normalization layer	125 × 94 × 256
C11, B11, R11	FS = 1 × 1, S = 1	125 × 94 × 64
C12, B12, R12	FS = 3 × 3, S = 1	125 × 94 × 64
C13, B13, R13	FS = 5 × 5, S = 1	125 × 94 × 64
C14, B14, R14	FS = 7 × 7, S = 1	125 × 94 × 64
CN3	Five inputs	125 × 94 × 512
B3x	Batch normalization layer	125 × 94 × 512
C15, B15, R15	FS = 1 × 1, S = 2	63 × 47 × 128
C16, B16, R16	FS = 3 × 3, S = 2	63 × 47 × 128
C17, B17, R17	FS = 5 × 5, S = 2	63 × 47 × 128
C18, B18, R18	FS = 7 × 7, S = 2	63 × 47 × 128
C27, B27, R27	FS = 3 × 3, S = 2	63 × 47 × 32
CN4	Five inputs	63 × 47 × 544
B4x	Batch normalization layer	63 × 47 × 544
C19, B19, R19	FS = 1 × 1, S = 1	63 × 47 × 256
C20, B20, R20	FS = 3 × 3, S = 1	63 × 47 × 256
C21, B21, R20	FS = 5 × 5, S = 1	63 × 47 × 256
C22, B22, R22	FS = 7 × 7, S = 1	63 × 47 × 256
C28, B28, R28	FS = 3 × 3, S = 4	63 × 47 × 32
C34, B34, R34	FS = 3 × 3, S = 2	63 × 47 × 32
CN5	Seven inputs	63 × 47 × 1632
B5x	Batch normalization layer	63 × 47 × 1632
C23, B23, R23	FS = 1 × 1, S = 2	32 × 24 × 384
C24, B24, R24	FS = 3 × 3, S = 2	32 × 24 × 384
C25, B25, R25	FS = 5 × 5, S = 2	32 × 24 × 384
C26, B26, R26	FS = 7 × 7, S = 2	32 × 24 × 384
C29, B29, R29	FS = 3 × 3, S = 8	32 × 24 × 32
C30, B30, R30	FS = 3 × 3, S = 2	32 × 24 × 32
C31, B31, R31	FS = 3 × 3, S = 4	32 × 24 × 32
C32, B32, R32	FS = 3 × 3, S = 4	32 × 24 × 32
C33, B33, R33	FS = 3 × 3, S = 8	32 × 24 × 32
CN6	Nine inputs	32 × 24 × 1696
B6x	Batch normalization layer	32 × 24 × 1696
G1	-	1 × 1 × 1696
F1	1200 FC (Fully Connected)	1 × 1 × 1200
D1	Dropout layer with learning rate: 0.5	1 × 1 × 1200
G2	2	1 × 1 × 2
O (softmax function)	benign, malignant	1 × 1 × 2

**Table 2:** The results of skin cancer classification on ISIC-2020.

Phases	Accuracy (%)	Recall (%)	Precision (%)	F-Score (%)
Phase #1	89.69	85.60	92.86	89.09
Phase #2	98.57	97.90	99.18	98.53

**Table 3:** The results of DFU classification.

Phases	Accuracy (%)	Recall (%)	Precision (%)	F-Score (%)
Phase #1	83.18	83.03	89.21	86.0
Phase #2	96.10	97.06	95.45	96.25
Phase #3	99.03	99.81	98.7	99.25

**Table 4:** Comparison of our result with the state-of-the-art results on DFU classification.

Method	Recall (%)	Precision (%)	F-Score (%)
DFUNet [61]	92.5	93.8	93.1
DFU_QUTNet [59]	92.6	94.2	93.4
DFU_QUTNet + KNN [59]	92.7	93.8	93.2
DFU_QUTNet + SVM [59]	93.6	95.4	94.5
Ours (Phase #3)	99.81	98.7	99.25

**Table 5:** Results of our model on the ICIAR-2018 dataset.

Phases	Image-Wise Accuracy (%)
Phase #1	85.29
Phase #2	97.51

**Table 6:** Comparison of our approach against the results of those from the state-of-the-art with the breast cancer classification scenario.

Phases	Image-Wise Accuracy (%)
Guo, Y., et al. [64]	87.5
Vang, Y.S., et al. [65]	87.5
Sarker, M.I., et al. [66]	89
Alzubaidi, L., et al. [67]	89.4
Ferreira, C.A., et al. [68]	90
Kassani, S.H., et al. [69]	92.5
Wang, Z., et al. [70]	93
Alzubaidi, L., et al. [14]	96.1
Ours (Phase #2)	97.51

## Data Availability

Code: https://github.com/muthanak/Novel-Transfer-Learning-for-Medical-image-classifiction (accessed on 23 March 2021).
